# Predictive Factor of the Possibility for Aortic Side Branches Coil Embolization during Endovascular Abdominal Aortic Aneurysm Repair

**DOI:** 10.3400/avd.oa.20-00115

**Published:** 2020-09-25

**Authors:** Atsushi Aoki, Kazuto Maruta, Norifumi Hosaka, Tomoaki Masuda, Tadashi Omoto, Yui Horikawa

**Affiliations:** 1 Department of Cardiovascular Surgery, Showa University Hospital; 2 Department of Radiology, Showa University Hospital

**Keywords:** abdominal aortic aneurysm, stent graft, embolization, inferior mesenteric artery, lumbar artery

## Abstract

**Objective**: Coil embolization of aortic side branches has been additionally performed to prevent type II endoleak during EVAR in our institute. In this study, we evaluated the predictive factors of the possibility for coil embolization of the inferior mesenteric artery (IMA) and lumbar artery (LA) during EVAR.

**Methods**: Seventy-four EVAR patients during June 2015 and April 2019 were included in the study. The coil embolization procedural time for one vessel is limited to 10 min. Aortic side branches were selected with 4 Fr Shepherd hook type catheter (Medikit, Tokyo, Japan) and were embolized with Interlock (Boston Scientific, MA, USA) via microcatheter. As predictive factors, internal diameter of aortic side branches and the aortic diameter perpendicular to the origin of LA (aortic diameter) were evaluated.

**Results**: Coil embolization was tried for 52 patent IMAs and all IMAs except two IMAs with ostial stenosis were successfully coil embolized (96.2%). Totally 190 LAs were patent and coil embolization was tried for 144 LAs. Among 144 LAs, 106 LAs (73.6%) were successfully coil embolized and the diameter was significantly longer (2.30±0.51 mm vs. 2.04±0.41 mm, p=0.007) and aortic dimeter was significantly shorter (30.0±8.1 mm vs. 40.5±11.6 mm, p<0.001) in successfully embolized LAs. Cut off value of successful LA coil embolization was 2.06 mm for internal diameter and 36.1 mm for aortic diameter by receiver operating characteristic curve analysis. Successful coil embolization rate for LAs with internal diameter longer than 2.0 mm and aortic diameter less than 36.2 mm was 90% (72 among 80 LAs).

**Conclusion**: Coil embolization during EVAR for IMA was highly successful, if there was no calcified ostial stenosis. LA embolization was feasible especially for LAs with internal diameter ≥2.0 mm and aortic diameter ≤36.1 mm. This information would be useful to select the target vessel for aortic side branches coil embolization during EVAR. (This is a translation of Jpn J Vasc Surg 2019; 28: 389–396.)

## Introduction

According to a report from the Japan Committee for Stent graft Management, the survival rate without the need for re-intervention for endovascular aneurysm repair (EVAR) was 83.3% during averaged follow up period of 2403 days.^1)^ Aortic aneurysms expanded 5 mm or more over 5 years in 23.3% patients and Type II endoleak (T2EL) is a risk factor for aneurysm enlargement.^1)^ In our previous study, inferior mesenteric arteries (IMA) and lumbar arteries (LA) with diameter of 2 mm or more were associated with T2EL observed on contrast-enhanced computed tomography (CT) at 7 days after surgery. Coil embolization was possible for 94% IMA and 64% LA without causing complications related to coil embolization, and the frequency of T2EL at 7 days after surgery decreased to 4% from 59%.^2)^ Presently, whether endovascular or open graft replacement for abdominal aortic aneurysm (AAA) repair, was selected based on the patient’s wish, the surgical risk, anatomical factors of the aneurysm. If coil embolization of the aortic branches during EVAR surgery is successful, the frequency of T2EL and the risk of aortic expansion will decrease. Thus, predicting whether coil embolization of the aortic branches is possible or not may influence treatment policy decisions. Furthermore, if coil embolization would be limited for the aortic branches with high possibility of successful coil embolization, the time required for coil embolization and the contrast agent usage would be reduced. Therefore, we examined whether it is possible to predict feasibility coil embolization.

## Materials and Methods

Among the patients who underwent EVAR from June 2015 to April 2019, 74 were included in this study after excluding cases of saccular aneurysm, iliac artery aneurysms, and infectious aneurysms. The mean age of the patients was 76.4 years and 11 were females (14.9%). The average preoperative aortic aneurysm diameter was 53.0 mm, and the operation time was 148±38 min. Patency of the aortic branches was evaluated using 0.7 or 1.0 mm slice contrast-enhanced CT before surgery, and when patented, the maximum diameter was considered as the branch diameter.

While all patent IMA and LA with internal diameters of at least 2 mm were targets of coil embolization, we also attempted embolization of the LA with the internal diameters were <2 mm when 4 or more LAs were patent. When the aortic diameter at the origin of the LA was smaller than the stent graft diameter, LA coil embolization was not attempted because the vessel would be occluded by the stent graft. Among the 74 patients, six did not have patent aortic branches and coil embolization was not performed, and coil embolization was attempted in only the IMAs because there were no patent LAs in 9 patients.

Technically, 4-Fr Shepherd hook catheters, RIM catheters, or KMP catheters (Medikit co. Ltd., Tokyo, Japan) were used to identify the aortic side branches. The IMA was selected from the right femoral artery, whereas the right LA was selected from the left femoral artery, and the left LA was selected from the right femoral artery. If identification was difficult, aortic branch selection was done by approaching from the opposite femoral artery. Once aortic side branch was identified, Renegade microcatheter (Boston Scientific, Marlborough, MA, USA) was inserted into the branches. After verification by angiography, embolization was performed using Interlock (Boston Scientific).

The IMA were accessed in anterograde fashion from within the sac. Coil embolization was always performed at the proximal part of the left colic artery. Coil embolization for the LA was performed at the proximal to the LA branch, so residual sac inflow from the LA branch was prevented. In cases the procedure was difficult due to kinking or tortuous iliac artery, 8-Fr long sheaths (Terumo Co., Tokyo, Japan) or 12-Fr Dryseal sheaths (W. L. Gore & Associated, Inc., Newark, DE, USA) were used.

All procedures were performed in a hybrid operating room equipped with a Siemens Artis Zeego (Siemens, Erlangen, Germany) and aortic branches were selected using fusion imaging.^2)^ From June 2018, we determined the location of the aortic branch origins corresponded to the frontal view of the lumbar vertebrae using multi-planar reconstruction images obtained from enhanced CT pre-operatively, and aortic branches were selected using the fluoroscopy image of the lumbar spine as an index during procedure. When selection was difficult, fusion imaging was also used. The procedural time for one target was limited up to 10 min. After 100 mL of contrast agent was used, coil embolization was abandoned. Coil embolization was considered to be successful when at least one coil could be placed in the aortic branches.

**Figure 1:**
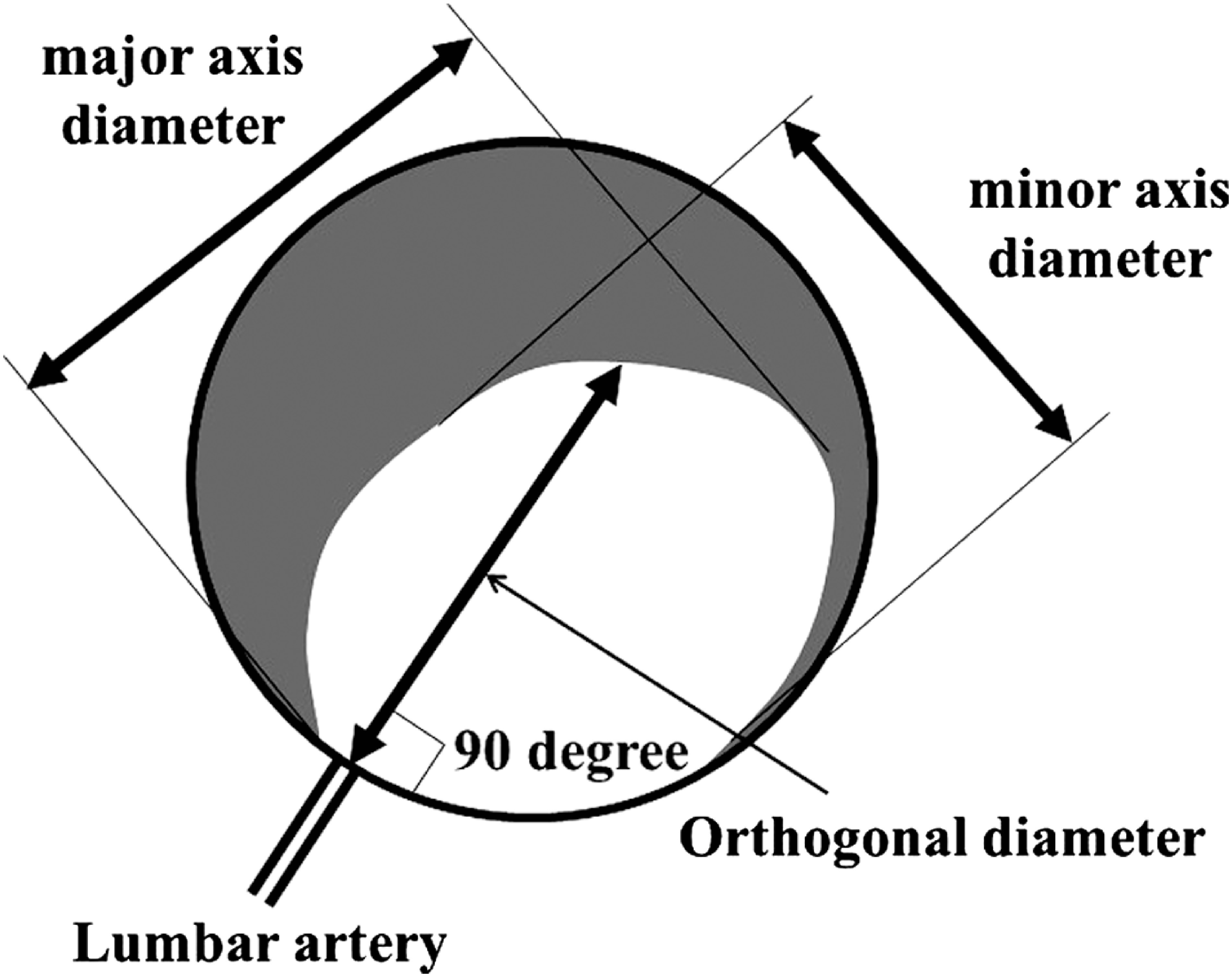
Fig. 1 Measurement of the aortic diameter.

To predict whether aortic branch coil embolization was possible, the inner diameter of the aortic side branches, minor and major diameters of the patent aorta at the origin of the side branch, and diameter of the patent aorta that is perpendicular to the aortic wall (orthogonal diameter) were evaluated (**Fig. 1**). For LA coil embolization, these factors were evaluated between successful coil embolized LAs (success group) and fail coil embolization LAs (failed group). The effectiveness of the method of using frontal view and lumbar vertebrae as an index was also evaluated.

Statistical analyses were performed using JMP Pro 13 software (SAS Inc., Cary, NC, USA). Chi-squared test or Fisher’s exact test were used for categorical variables. Wilcoxon test was used for continuous variables. Cut-off values for predictors were estimated with receiver operating characteristic curves (ROC curve) using the Youden index.

## Results

Patent IMA was present in 52 of 74 cases (70.3%) and coil embolization was attempted in these 52 cases. Coil embolization was possible in all patients except 2 patients (96.2%). The two cases in which coil embolization was not possible had inner vessel diameters of 1.89 and 2.39 mm, and each vessel had calcification and stenosis at those origin. Although micro-guidewire insertion was achieved in these two cases, the Renegade microcatheter could not be inserted and coil embolization was abandoned. In both these 2 cases where coil embolization was not possible, contrast-enhanced CT 7 after the procedure revealed IMA occlusion, and T2EL from IMA was not observed. To select the IMA, Shepherd hook (70.0%), KMP (22.0%), and RIM type (8.0%) catheters were used.

According to the LAs, 260 out of 450 LAs were occluded by preoperative CT. **Table 1** shows the number of patent LAs based on the lumbar vertebrae height and left or right, reasons why embolization was not attempted, number of LAs for which embolization was attempted, and the success rate for coil embolization. In a majority of cases, the third and fourth LAs were embolized. No significant differences were observed for success rates between lumbar vertebrae heights or right and left. Coil embolization was not attempted for 31 out of 190 patent LAs because of narrow internal diameters of 0.90 to 1.86 mm, with a mean of 1.35 mm. For 15 LAs, coil embolization was not attempted because the LAs origin would have been covered by the stent graft. Therefore coil embolization was attempted for 144 LAs and coil embolization was possible for 106 (73.6%) LAs (success group), whereas the procedure was failed for 38 LAs (failed group). There were nine patent median sacral arteries and four had narrow diameters of 0.94 to 1.51 mm and an average of 1.24 mm. Coil embolization was attempted for five, and the procedure was possible for three (60%) vessels.

**Table 1:** Table 1 Demographics of neglected/targeted lumbar arteries

The sides and heights to the lumbar vertebrae	Patent lumbar artery (n)	<2 mm LA (embolization not attempted)	LA covered by device (embolization was not attempted)	Number of LA coil embolization was attempted (percentage for patent LA)	Number of success/failed coil embolization (success rate of coil embolization)
Third, right	48	10	5	33 (68.8%)	25/33 (75.8%)
Third, left	46	5	7	34 (73.9%)	26/34 (76.5%)
Forth, right	37	7	1	29 (78.4%)	21/29 (72.4%)
Forth, left	41	4	1	36 (87.8%)	26/36 (72.2%)
Fifth, right	10	3	0	7 (70.0%)	5/7 (71.4%)
Fifth, left	8	2	1	5 (62.5%)	3/5 (60.0%)
Middle sacral artery	9	4	0	5 (55.6%)	3/5 (60.0%)

Coil embolization was attempted in 56–88% of patent lumbar arteries depend on the sides and heights to the lumbar vertebrae. Success rate of coil embolization of sides and heights to the lumbar vertebrae did not differ significantly. For twelve out of 94 lumbar arteries of third vertebrae, coil embolization was not attempted because the aortic diameter at its origin was smaller than the diameter of stentgraft and lumbar artery would be closed by stentgraft.LA: Lumbar artery

Fluoroscopic time during the procedure was 15–162 (mean, 60±28) min, radiation dose was 0.14–7.15 (mean, 1.38±1.12) mGy, and volume of contrast agent used was 62–445 (mean, 191±75) mL. Renal function was evaluated excluding patients on chronic dialysis. Pre-operative serum creatinine level was 0.91±0.22 mg/dL and serum creatinine level decreased to 0.83±0.21 (p<0.001) on 3 days after procedure, and to 0.85±0.23 mg/dL (p<0.001) on 7 days after procedure. The estimated glomerular filtration rate (eGFR) values were 64.0±18.9 mL/min/1.73 m^2^ pre-operatively and increased to 70.9±20.0 mL/min/1.73 m^2^ (p<0.001) and 3 days after procedure, and to 68.8±23.3 mL/min/1.73 m^2^ (p=0.002) 7 days after procedure. On day 3 after surgery, an increased creatinine level of ≥0.5 mg/dL or an increase of ≥25% from the preoperative values was observed in only one patient. This patient was 85-year-old woman with pre-operative serum creatine level was 0.99 mg/dL and eGFR was 40.5 mL/min/1.73 m^2^, and the amount of contrast agent was 62 mL.

**Figure 2:**
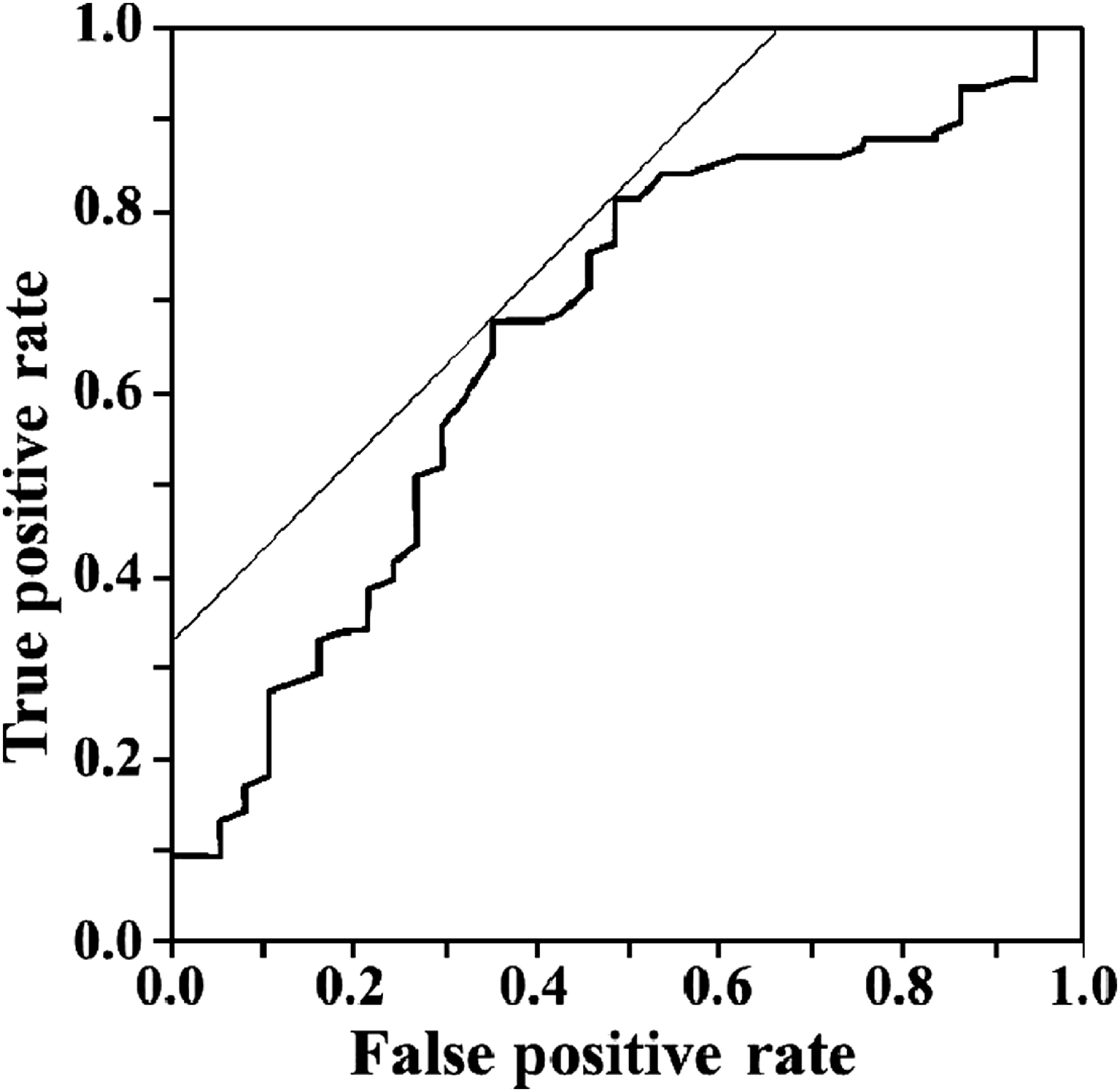
Fig. 2 ROC curve of internal diameter of the lumbar artery for success or failure of coil embolization.

**Table 2** shows the types and branches for which embolization was attempted as well as the number of patients, fluoroscopic time, radiation dose, and the amount of contrast agent. The fluoroscopic time, radiation dose, and contrast agent use in the six cases for which coil embolization was not attempted (no-embolism group) were compared to the 28 cases with 1–2 coil embolization (1–2 group) and the 36 cases with at least three embolization (≥3 group). The fluoroscopic times were 33±19, 49±16, and 72±30 min for the no-embolism, 1–2, and ≥3 or more groups, respectively. The fluoroscopic time in the 1–2 group tended to be longer than the no-embolism group (p=0.082), whereas the ≥3 group had significantly longer fluoroscopic times than the no-embolism (p=0.003) and 1–2 groups (p<0.001). The radiation doses were 0.49±0.42, 1.02±0.78, and 1.83±1.24 mGy in the no-embolism, 1–2, and ≥3 groups, respectively. Significantly higher radiation doses were used in the 1–2 group than the no-embolism group (p=0.021), whereas the ≥3 group used significantly higher doses than the no-embolism (p<0.001) and 1–2 groups (p<0.001). The amount of contrast agent was 125±83, 166±56, and 221±75 mL in the no-embolism, 1–2, and ≥3 groups, respectively. The 1–2 group tended to use more contrast than the no-embolism group (p=0.082), whereas the ≥3 group used significantly more contrast than the no-embolism (p=0.011) and 1–2 groups (p=0.002). Prior to May 2018, 41 of 59 vessels (69.5%) in the first set of 29 cases could be embolized, whereas 31 of 44 vessels (70.5%) in the second set of 28 cases were embolized, with no significant differences in the success rates between the first and second sets (p=0.916). Comparing the success and failed groups, the LA luminal diameters were significantly larger in the success group (2.30±0.51 mm) than in the failed group (2.04±0.41 mm; p=0.0069). To decide whether coil embolization will be successful, the cut-off value for the luminal diameter, determined by ROC curve analysis, was estimated to be 2.06 mm (**Fig. 2**).

**Table 2:** Table 2 Patterns of branch embolization and results

Patterns of branch embolization	Number of patients (%)	Fluoroscopic time (min)	Radiation dosage (mGy)	Amount of contrast agent (mL)
None	6 (8.1%)	33±19	0.49±0.42	125±83
1 LA	6 (8.1%)	55±24	0.82±0.39	175±93
2 LAs	4 (5.4%)	40±18	1.26±0.69	179±53
3 LAs	2 (2.7%)	107	1.59	316
4 LAs	3 (4.1%)	84±45	3.83±2.92	313±115
5 LAs	1 (1.4%)	96	1.44	232
IMA only	9 (12.2%)	45±14	0.79±0.32	173±28
IMA+1 LA	11 (14.9%)	52±12	1.18±1.13	153±49
IMA+2 LAs	13 (17.6%)	76±30	1.67±0.90	231±74
IMA+3 LAs	10 (13.5%)	61±17	1.43±0.55	189±49
IMA+4 LAs	7 (9.5%)	63±23	1.63±0.95	181±44
IMA+5 LAs	2 (2.7%)	70	2.46	214

In 6 patients, no coil embolization was attempted and only IMA coil embolization was tried in 9 patients. When the patients were divided into none, 1–2 and 3 or more depend on the number of coil embolization attempted aortic side branches, fluoroscopic time tended to be longer in 1–2 than none and was significantly longer in 3 or more than none and 1–2. Radiation dosage was significantly larger in 1–2 than none, and significantly larger in 3 or more than none and 1–2. The amount of contrast agent was tended to be larger in 1–2 than none, and significantly larger in 3 or more than none and 1–2. LA: Lumbar artery, IMA: Inferior mesenteric artery

**Figure 3:**
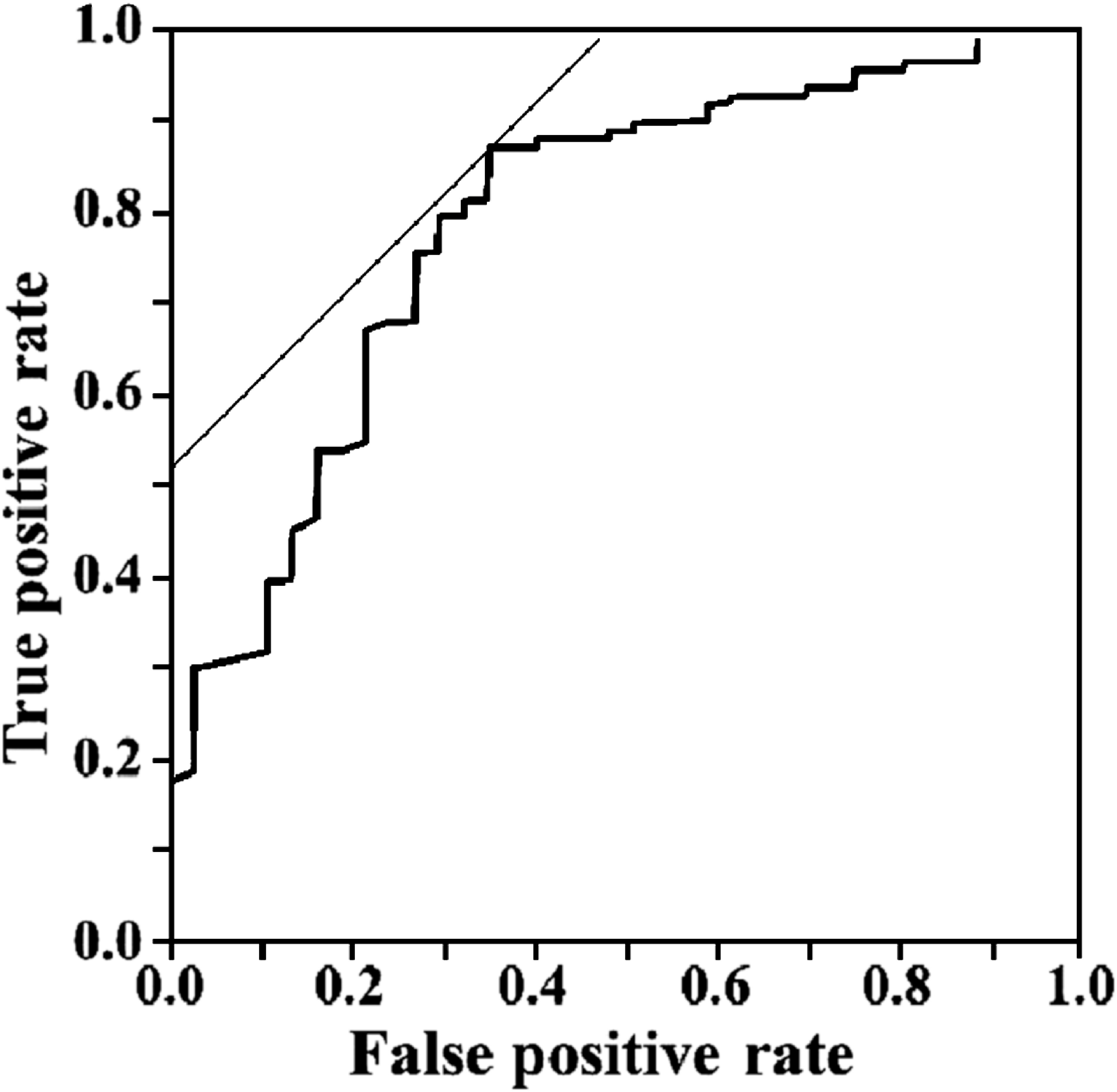
Fig. 3 ROC curve of orthogonally crossing diameter of the aorta at the level of lumbar artery orifice for success or failure of coil embolization.

Regarding the inner aortic diameters, the short axis diameters were 29.0±8.9 and 37.6±13.2 mm in the success and failed groups, respectively (p=0.003), and the long axis diameters were 35.2±8.8 and 40.3±13.9 mm in the success and failed groups, respectively (p=0.0043). Further, the orthogonal diameters were 30.0±8.1 and 40.5±11.6 mm in the success and failed groups, respectively (p<0.0001). All diameters were significantly smaller in the success group than in the non-success group, with the orthogonal diameter having the lowest p-value. The cut-off value of the orthogonal diameter for successful coil embolization was 36.1 mm (**Fig. 3**). Of the 80 LAs with inner diameters of ≥2 mm and orthogonal diameters of ≤36.1 mm, coil embolization was possible for 72 vessels (90%).

The LA embolization success rate was 72 of 103 (69.9%) before introducing a method of selecting LA using the position of the lumbar vertebrae in the front view. After introduction of this selection method, the success rate was 34 of 41 (82.9%). No significant difference was observed (p=0.0994) in success rates before and after introducing this selection method.

## Discussion

As EVAR is minimally invasive procedure, its use has rapidly increased in Japan after the introduction of manufactural stent grafts in 2007. However, more than 10 years have been passed since EVAR introduction, it became apparent that aneurysm sac expanded in about a quarter of cases within 5 years after EVAR.^1)^ T2EL is a risk factor for aneurysm enlargement.^1)^ Many risk factors for T2EL have been identified including patient background and aneurysm morphology. Advanced age and female sex are risk factors for T2EL. A history of smoking or peripheral vascular disease decreases the risk of T2EL.^3)^ The risk of T2EL increases when the maximum aneurysm diameter is large,^4)^ but decreases when there is a circumferential thrombus.^3)^ Because modification of these factors to reduce T2EL would be difficult, we evaluated the aortic side branched which the cause of T2EL. According to our results, the presence of patent IMAs with diameters of ≥2.5 mm, many patent lumbar arteries, or patent LAs with luminal diameters of ≥2.0 mm are associated with T2EL.^2)^ These results are consistent with previous studies where the following were associated with increased T2EL frequency: patent IMA,^3,5–9)^ especially with a diameter of ≥2.5 mm^3,7,10)^; vessel with a large diameter of ≥3 mm^11,12)^; many patent LAs,^4,6,8,10,11)^ especially four or more^8,12)^; and LA lumen diameter of ≥1.9–2.0 mm.^3,4,9)^

When the aneurysm sac expansion occurs due to T2EL, various approaches are used to embolize or ligate the culprit vessel. In a 2018 meta-analysis^13)^ conducted by Ultee et al., the technical success rate, which means no blood flow into the aneurysm sac during procedure was 87.9%, whereas the clinical success rate, which means disappearance of T2EL or the aneurysm sac shrinkage during follow-up, was as low as 68.4%. Thus secondary intervention for T2EL would be safe, however there is little evidence of the usefulness of secondary intervention for T2EL. According to histological studies, the arterial wall enlarged by T2EL is more vulnerable than the arterial wall of a normal aorta or that of an abdominal aortic aneurysm before treatment,^14)^ and cases requiring open abdominal surgery for the aneurysm sac expansion due to T2EL have increased.^15)^ Once the aneurysm sac enlargement occurs due to T2EL, treatment becomes difficult; thus, preoperative or intraoperative embolization of the aortic branches has been performed since 2002.^16)^ The risk of aneurysm expansion increases when the contrast enhanced area or volume by T2EL is large.^17,18)^ T2EL due to IMA is considered to have a large contrast enhanced area,^10)^ with a greater effect on aneurysm expansion. Moreover, T2EL caused by IMA and LA (IMA-LA T2EL) are risk factors for aneurysm expansion.^19)^ Therefore, a patent IMA is thought to be an important cause of T2EL. Many studies^10,16,20–27)^ have reported preoperative or intraoperative embolization attempts for the IMA and the success rate has been reported as ranged from 88.7%–100%. In these reports, embolization was attempted for 457 IMAs in total, with a remarkably high success rate of 95.8% has been reported. There was only one case of colon necrosis occurring as a complication of IMA embolization.^22)^ This case is special because the patient had a history of expanded right hemicolectomy including the resection of middle colic artery and the patient developed colon necrosis and subsequently underwent left hemicolectomy after IMA embolization. Thus, IMA embolization is thought to be relatively safe and can be performed at a high success rate. However, T2EL from LA may occur when only IMA embolization is performed and T2EL from LA occurs in 14%–34% cases in these reports. In studies embolizing both the IMA and LA,^10,16,20,23,24)^ the success rate of LA embolization was 62%–92%. However, for studies that showed the actual number of vessels with embolization attempts,^10,16,20,24)^ the success rate was 69.0% for 116 attempts. The frequency of T2EL after IMA and LA embolization reported in these studies was extremely low, with values of 0%–4.5%. Therefore, the T2EL preventive effect is greater when the IMA as well as for the aortic LA branches with diameters of at least ≥2 mm were embolized. The low frequency of embolization for the LA is due to the technical difficulty related to the meandering of the LA from the aortic wall, renal dysfunction due to increased contrast agent used, and increased radiation exposure. In our study, when the number of branches for target coil embolization increased, fluoroscopy time was extended, and the radiation dose and the amount of contrast agent increased. Therefore, if the aortic branches with a high success rate of embolization can be identified and targeted, the amount of radiation exposure and contrast agent might be reduced. Furthermore, the prediction of high success rate of coil embolization of aortic branches, which reduces the risk of T2EL, may influence in deciding between EVAR and open repair with artificial graft replacement for treating AAA. Therefore, we examined the factors that affect the success rate of embolization of aortic side branches and tried to identify the indices that help select the side branches for coil embolization preoperatively.

If the origin of the IMA is not stenotic, embolization was possible in all cases. Fukuda et al.^7)^ reported that an IMA with stenosis at its origin becomes occluded postoperatively and was unlikely to cause T2EL. In our study, embolization was not possible for two IMAs with stenosis at their origins, however contrast-enhanced CT 7 days after procedure revealed IMA occlusion, suggesting that if IMA is stenotic at the origin, the possibility of postoperative T2EL due to IMA would be low. The Shepherd hook-type catheter was used for IMA selection with a frequency of 80%. The tip of the Shepherd hook catheter faces the peripheral side. Therefore, the IMA cannot be selected by the Shepherd hook catheter if the IMA originates from the vicinity of the transition from the proximal neck to the aneurysm. The KMP-type catheter may be useful in this situation.

Regarding LA, the orthogonal aortic diameter and inner diameters of the LA are predictors of successful coil embolization. The orthogonal diameter is a predictor because the tip of the 4-Fr Shepherd hook catheter cannot reach to the aneurysm wall if the inner diameter is large, making it impossible for LA selection.

This study demonstrated that coil embolization of aortic side branches can be performed at a high success rate for IMA during EVAR. For the LA, coil embolization can be performed at a high success rate if the orthogonal aortic diameter is less than 36 mm. In 2018, a 4 dimensional-flow magnetic resonance imaging method of identifying branches causing T2EL aneurysm enlargement after EVAR surgery^28)^ was reported. In the future, methods to identify the aortic branches that cause T2EL prior to EVAR should be developed. If the identification of aortic branches that case T2EL is not possible, the extent of LA coil embolization required to prevent T2EL should be examined.

In this study, coil embolization of aortic branches was performed in a hybrid operating room where fusion imaging can be used. When the angle is set to see the aortic branch origin laterally in the fusion image, the actual branch origin shifts about 4–5 mm dorsally or ventrally if the fluoroscopic angle deviates by 10 degrees in the aortic aneurysm with 40–50 mm diameter, making its selection difficult. When the LA is selected using the fluoroscopy image of the lumbar vertebrae in the frontal view, the dorsal-facing catheter may be operated from two dimensions and aortic branches may be identified. In our experience, this selection method also allows the selection of aortic branches at the same frequency as using fusion images. Even in mobile angiography devices, if the LA diameter is ≥2 mm and the inner diameter of the aorta is ≤36 mm, it may be possible to perform LA coil embolization.

As this is a single-center study, the factors associated with successful coil embolization of aortic side branches should be verified in other facilities. We performed coil embolization of aortic branches during EVAR, and there is a limit for the procedure time and the amount of contrast agent. However, if the coil embolization is performed in the angiography room preoperatively and EVAR is performed in a two-stage manner, successful coil embolization of aortic branches is possible. This should be the subject of future investigations.

In this study, coil embolization of aortic branches was investigated, but another method of reducing T2EL involves promoting thrombus formation in the aneurysmal sac after EVAR. In 2007, Zanchetta et al.^29)^ injected fibrin glue into the aneurysm sac after EVAR and reported a decrease in T2EL. In 2010, the same group^30)^ reported a significant decrease in T2EL by sac “thrombization.” Similarly, in 2016, Piazza et al.^11)^ conducted a trial in patients at high risk for T2EL (with a patent IMA of diameter ≥3 mm, three or more pairs of patent LA, two patent LA pairs plus a patent medial sacral artery, or patent IMA). One hundred and seven patients who were high risk for T2EL were randomized to receive standard EVAR or EVAR with intraoperative embolization by fibrin glue injection and coil placement depending on the volume of the aneurysm sac (sac embolization group). T2EL decreased after three and six months and frequency of T2EL-related secondary intervention decreased over 2 years in the sac embolization group. However, in the report by Ronsivalle et al.,^30)^ one patient had colon ischemia, and underwent colorectal resection among 121 “thrombization” patients. There are no reports on complications associated with LA coil embolization, and in our experience, no case of spinal cord injury, colon ischemia, or muscle necrosis resulting from coil embolization was encountered. We believe that coil embolization of aortic branches can be safely performed.

## Conclusion

Intraoperative coil embolization of aortic branches during EVAR to prevent T2EL was highly possible for an IMA not having stenosis at its origin. For the LA, embolization was possible in 70% of the vessels, and if the diameter of the patent aorta perpendicular to the LA origin was ≤36.1 mm and the LA inner diameter was ≥2 mm, coil embolization was possible at a frequency of 90%.

## Additional Note

Presented at the 47th Annual Meeting of the Japanese Society of Vascular Surgeons (Nagoya, May 23, 2019).

## Notes

This is a translation of Jpn J Vasc Surg 2019; 28: 389–396.
